# Genome Sequences of *Salmonella enterica* subsp. *enterica* Serovar Lubbock Strains Isolated from Liver Abscesses of Feedlot Cattle

**DOI:** 10.1128/genomeA.00319-16

**Published:** 2016-05-05

**Authors:** Raghavendra G. Amachawadi, Milton Thomas, Tiruvoor G. Nagaraja, Joy Scaria

**Affiliations:** a Department of Diagnostic Medicine and Pathobiology, College of Veterinary Medicine, Kansas State University, Manhattan, Kansas, USA; b Department of Veterinary and Biomedical Sciences, South Dakota State University, Brookings, South Dakota, USA

## Abstract

The genome sequencing of 13 *Salmonella enterica* subsp. *enterica* serovar Lubbock strains isolated from liver abscesses of feedlot cattle is reported here. The availability of these genomes will help to further understand the etiologic role of *Salmonella* strains in liver abscesses of cattle and will serve as references in microbial trace-back studies to improve food safety.

## GENOME ANNOUNCEMENT

Liver abscesses occur in feedlot cattle as a consequence of feeding them a high-grain diet (1). Cattle with severely abscessed livers have lower feed intake, reduced weight gain, and a decreased gain-to-feed ratio (2). The primary causative agent of liver abscess is *Fusobacterium necrophorum* (3). The ruminal acidosis resulting from the highly fermentable starch contained in the grains, and subsequent rumenitis, facilitate the migration of *F. necrophorum* from the rumen to the liver via portal circulation (1). Recently, for the first time, we reported the occurrence, along with *F. necrophorum*, of a novel *Salmonella* serotype, designated 6,7:g,m,s:e,n,z15, now named *Salmonella enterica* subsp. *enterica* serovar Lubbock (4), in liver abscesses of cattle (5). The newly reported serotype *S.* Lubbock is closely related to *S. enterica* subsp. *enterica* serovar Mbandaka and has been isolated from subiliac lymph nodes of healthy cattle (4). It is not known whether *S.* Lubbock is a causative agent of liver abscesses or is a secondary invader, via or lymph or blood, of an abscess initiated by *F. necrophorum*. In a recent study, we observed that *Salmonella* was prevalent in 20 to 25% of the abscesses cultured, and *S.* Lubbock was the predominant serotype. Here, we report the availability of draft genomes of 13 *S.* Lubbock strains isolated from liver abscesses.

*S.* Lubbock strains were isolated from liver abscesses of feedlot cattle, as per a previously described protocol (5). The serotypes of the isolates were determined at the National Veterinary Service Laboratory (NVSL), Ames, Iowa. Strains were grown in brain heart infusion broth for 12 h at 37°C. DNA from each strain was isolated from 1.0-ml cultures using the E.Z.N.A. bacterial DNA kit (Omega Bio-tek, Norcross, GA). According to the manufacturer’s protocol, sequencing libraries were prepared using 1.0 ng of genomic DNA using the Nextera XT kit (Illumina, San Diego, CA). We used V2 paired-end chemistry (2 × 250 bp) to sequence the genomes on an Illumina MiSeq platform. *De novo* genome assembly was performed using SPAdes version 3.5.0 (6), available at http://bioinf.spbau.ru/spades. Genome annotation was performed using the NCBI Prokaryotic Genome Automatic Annotation Pipeline (PGAAP) (7).

The genome characteristics of the 13 *S.* Lubbock strains are summarized in Table 1. The serotypes of the 13 strains were confirmed using SeqSero (8). Genome size and G+C content were estimated for all contigs of each strain. Among the 13 strains, the median values for genome size and G+C content were 4.97 Mb and 52.1%, respectively (Table 1), and were similar to those of previously published *S. enterica* genomes.

**TABLE 1:** Characteristics of 13 *S.* Lubbock strains isolated from liver abscesses of cattle

Strain name	GenBank accession no.	Genome size (bp)	G+C content (%)	Total no. of contigs
LA-10-2013	LSMA00000000	4,973,701	52.1	128
LA-1-2013	LSLN00000000	4,955,079	52.1	100
LA-2-2013	LSLO00000000	4,959,869	52.1	108
LA-3-2013	LSLP00000000	4,988,702	52.1	174
LA-4-2013	LSLQ00000000	4,964,148	52.1	112
LA-5-2013	LSLR00000000	5,174,970	52.1	479
LA-5-2014	LSLS00000000	5,032,588	52.1	267
LA-6-2013	LSLT00000000	4,983,284	52.1	184
LA-7-2013	LSLU00000000	4,992,701	52.0	175
LA-7-2014	LSLV00000000	4,979,081	52.1	142
LA-8-2013	LSLW00000000	4,979,081	52.1	142
LA-8-2014	LSLX00000000	4,961,787	52.1	106
LA-9-2014	LSLZ00000000	4,870,086	52.1	159

The availability of the genomes of 13 *S.* Lubbock strains is the first report of this serotype isolated from liver abscesses of cattle. The availability of these genomes will help to further understand the etiologic role of *Salmonella* strains in liver abscesses in cattle and will serve as references in microbial trace-back studies to improve food safety.

### Nucleotide sequence accession numbers.

The sequences have been deposited as whole-genome shotgun projects at GenBank under the accession numbers listed in Table 1.
